# Male soldiers are functional in the Japanese strain of a polyembryonic wasp

**DOI:** 10.1038/srep02312

**Published:** 2013-07-30

**Authors:** Daisuke Uka, Azusa Takahashi-Nakaguchi, Jin Yoshimura, Kikuo Iwabuchi

**Affiliations:** 1 Faculty of Agriculture, Tokyo University of Agriculture and Technology, Saiwai-cho, Fuchu, Tokyo 183–8509, Japan; 2 Graduate School of Science and Technology and Department of Systems Engineering, Shizuoka University, 3-5-1 Johoku, Naka-ku, Hamamatsu, 432–8561, Japan; 3 Marine Biosystems Research Center, Chiba University, Uchiura, Kamogawa, Chiba 299–5502, Japan; 4 Department of Environmental and Forest Biology, State University of New York College of Environmental Science and Forestry, Syracuse, NY 13210 USA; 5 Current address: Medical Mycology Research Center, Chiba University, 1-8-1 Inohana, Chuo-ku, Chiba 260–8673, Japan.

## Abstract

Polyembryonic parasitoids clonally produce sterile soldier larvae in both sexes. Female soldier larvae of *Copidosoma floridanum* defend their siblings and host resources against heterospecific competitors as well as conspecific male embryos that results in female biased sex ratios. However, the male soldiers of the USA strain exhibit no aggressive behaviors against them, suspected to be a secondary loss of male defense function in the course of evolution. From vitro and vivo experiments, we have found functional male soldiers in the Japanese strain of *C. floridanum*. In vitro experiments, male soldiers exhibit aggressions against four larval competitors, though aggressiveness is much weaker than that of female soldiers. In vivo experiments, heterospecific competitors are equivocally excluded in both male and female broods. Our findings support the idea that male soldiers have evolved primarily to defend against heterospecific competitors. Further experiments against conspecific embryos may be able to confirm this hypothesis.

Sterile female caste is most conspicuously observed in many hymenopteran insects that had evolved eusociality, e.g., bees, wasps and ants. However, in hymenopteran insects, sterile male caste is only known in polyembryonic parasitic wasps. Male caste formation in these polyembryonic parasitoids has been reported well in the genus *Copidosoma* and other closely related Encyrtid genera^1^,^2^. These species produce larval soldiers in both sexes. A previous study using *C. floridanum* from North America has demonstrated that the female soldier larvae exhibit aggression toward conspecific and heterospecific competitors^1^,^3^,^4^, but the male soldiers are not aggressive at all^5^. These male soldiers are very similar to female soldiers in their structurally distinctive slender forms, but exhibit no defensive behaviors against the competitors. Thus, these male soldiers are identified as (or called) “nonfunctional soldiers.”

In the female brood, the production of female soldiers is explained by at least two functions (hypotheses): (1) the defense against heterospecific competitors, and (2) sex ratio adjustment in a mixed brood, where male embryos are attacked and killed by female soldiers to promote female biased sex ratios. The above two hypotheses are not exclusive each other. However, in the male brood, these hypotheses cannot be used because nonfunctional male soldiers neither attack heterospecific competitors, nor the own reproductive embryos in a mixed brood. One hypothesis for male soldiers is the sex-ratio conflict, in which the production of nonfunctional male soldiers promotes female biased sex-ratio at emergence in a mixed brood^1^,^4^,^5^,^6^. This is a passive variation of the second sex-ratio hypothesis in female soldiers. However, this hypothesis cannot explain why the host resource is used for the nonfunctional male soldiers, instead of reducing the number of male reproductive embryos. Another hypothesis is the secondary loss of defensive behaviors in the course of evolution^5^. However, we currently have no enough data to support either hypothesis.

The previous studies of *C. floridanum* indicate that there are differences between the populations originated from the USA and Japan (we here call the USA and Japanese strains). In the USA strain, the development of soldier larvae differs between the sexes^1^,^7^,^8^. That is, female soldiers can be found in first-instar hosts, and then increase their number with host development up to two hundreds. In contrast, in males, only a few soldiers develop late in the host development. In contrast, in the Japanese strain, there is no sexual difference in soldier development^9^. That is, male and female eggs produce similar number of soldiers, in which soldier larvae of both sexes first appear at 1st or 2nd host instar and then increase their number up to more than one hundred. This sexual developmental difference between the USA and Japanese strains may reflect the differences of the functionality of male solders in these two strains. We suspect that male soldiers should have evolved to be functional at the first place (possibly the Japanese strain) and the USA strain might have lost its functionality. We therefore evaluate the functionality of male soldiers in the Japanese strain by *in vitro* contests and *in vivo* assessments. This finding should contribute to our questions on the evolution of male soldier castes: why they are very rare; why only found in polyembryonic wasps in hymenopteran insects.

## Results

### In vitro assay

**Figure 1:**
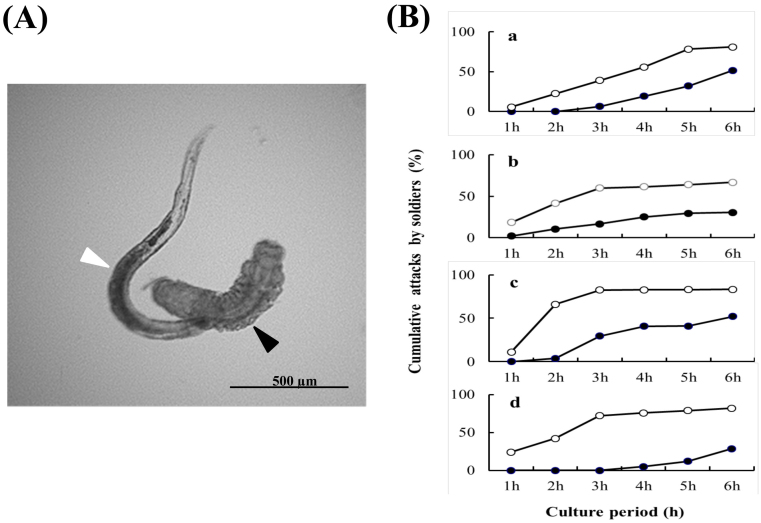
Defense behavior of *C. floridanum* male soldier. (A) A male soldier (white arrowhead) attacking a *G. pallipes* larva (black arrowhead). The image was obtained with a fluorescence microscope (BZ-9000, Keyence, Osaka, Japan). (B) Percentage of cumulative attacks by the soldiers of both sexes in *in* *vitro* contests with competitor parasitoid larvae: (a) *G. pallipes*, (b) *C. ruficrus*, (c) *M. pulchricornis*, (d) *C. glomerata*. 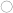 female soldiers, 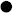 male soldiers.

In the Japanese strain of *C. floridanum*, *in vitro* contests revealed that the male soldiers exhibited aggressive behavior toward heterospecific parasitoid larvae, as in the female soldiers (Fig. 1). The *in vitro* contests revealed that soldiers of both sexes actively attacked the larvae of the other parasitoid species (Fig. 1A). The larvae being attacked all contracted their bodies and died within several minutes. Although both male and female soldiers exhibited aggressive behavior towards the larvae of potential parasitoid competitors, male soldiers are lower in attacking rates (Fig. 1B) and the time till attack is also longer (Table 1). For example, significantly fewer male soldiers attacked their opponents during the test period than female soldiers (against *G. pallipes*, male soldier = 51.6%; female soldier = 81.1%; *χ^2^* = 6.7579, *df* = 1, *P* = 0.009, Fig. 1B(a); against *C. ruficrus*, male soldier = 30.6%; female soldiers = 66.7%; *χ^2^* = 8.911, *df* = 1, *p* = 0.0028, Fig. 1B(b); against *M. pulchricornis*, male soldier = 52.0%; female soldier = 83.3%; *χ^2^* = 4.703, *df* = 1, *p* = 0.0301, Fig. 1B(c); against *C. glomerata*, male soldier = 28.6%; female soldier = 81.8%; *χ^2^* = 7.00, *df* = 1, *p* = 0.00005, Fig. 1B(d)).

In all cases, the male soldiers required a longer time till attack (against *G. pallipes*, t = 2.08, *df* = 21, p = 0.038; *C. ruficrus*, t = 2.08, *df* = 22, p = 0.023; *M. pulchricornis*, t = 2.12, *df* = 16, p = 0.0003; *C. glomerata*, t = 2.05, *df* = 25, p = 0.0001).

These results indicated that *C. floridanum* soldiers of both sexes exhibited aggression towards heterospecific competitor larvae and eventually killed them. In addition, these results also suggested that male soldiers were less aggressive than female soldiers, because of their tendencies to attack less frequently.

### In vivo contest

To validate that the aggressive behavior observed *in vitro* reflects the *in vivo* situation, we examined the competitive ability of both sexes against three natural parasitoids used for *in vitro* contests. When the hosts were multiparasitized by *C. floridanum* (the Japanese strain) and one of the natural parasitoids, adults of only one species emerged from each host. In all cases, *C. floridanum* adults were produced in significantly greater numbers than the other parasitoids (Table 2). Furthermore, there were no significant differences between the sexes of *C. floridanum* (Fisher's exact probability test, P = 0.1203 against *G. pallipes*, n = 100; P = 0.7479 against *C. ruficrus*, n = 100; P = 0.4945 against *M. pulchricornis*, n = 50), suggesting that both sexes of *C. floridanum* were competitively superior to the other natural parasitoids tested.

### Field survey of sex ratios in mixed broods

The male ratios of mixed broods are extremely biased to females averaging less than 0.06 for all four years examined (Table 3).

## Discussion

*In vitro* contests showed that the male soldiers of the Japanese strain exhibited aggressive behavior toward heterospecific parasitoid larvae, as in the female soldiers (Fig. 1, Table 1). This result coincided with the result of *in vivo* assessment (Table 2). These findings suggest that a functional sterile caste in larval males could have also developed as an adaptive response to competitors, as in larval females. The current result with the Japanese strain of *C. floridanum* is a sharp contrast to the report from the USA strain, in which male soldiers lack apparent adaptive function^5^.

The importance of this finding is that both functional and nonfunctional sterile male soldiers are found in *C. floridanum*, even though the difference in the functionality of male soldiers may not reflect the difference between the two geographical strains. Furtheremore, the current difference in male functionality between the two strains is also reflected in the developmental stages of male soldiers. The functional Japanese male soldiers are developed earlier in the host stages, while the nonfunctional USA male soldiers only so in later host stages. This fact strongly suggests the “secondary-loss hypothesis” of male functions^5^. In a closely related *C. kohleri*, in which only ca. 50 adults emerges from a single host, no male soldiers are produced^10^. The evolution of male soldiers may be limited by some ecological conditions, such as sex ratios and intraspecific competition^11^. The evolution of male functionality may be suppressed by intraspecific competition^6^,^11^.

We should also note that the developmental schedule of soldiers is likely to be genetically controlled. The number of male soldiers per host is a highly heritable trait and varies among clones^12^. The timing of emergence of the first soldier larvae that are specialized to attack conspecific competitors is also genetically determined^13^.

Our *in vitro* study also showed that there are sexual differences in the level of aggressiveness of soldiers. While the attack frequency increases with culture periods, those of female attacks are always considerably higher than those of males in all periods in all competitor species (Fig. 1B). In a related polyembryonic species *Copidosoma bakeri*, which produces mostly single-sex broods, the soldiers of both sex are shown to attack heterospecific parasitoid larvae^2^.

However, a *C. floridanum* female frequently lays one fertilized egg (being female wasps) and one unfertilized egg (being male wasps) in the same host. Furthermore, when one female lays only a fertilized (female) egg of a host, the second female tends to lay an unfertilized (male) egg on this host. In these mixed broods, only a few males always emerge from the host, but assure the copulations for all the emerging females from the same host^1^,^3^,^12^. Thus the copulation success of both male and female wasps emerging from mixed broods is guaranteed. In these mixed broods, functional male soldiers become non-adaptive or even maladaptive, if they attack reproductive female embryos that are the future mates of their own male broods^2^,^5^. This problem of conspecific attack is evident in the Japanese strain, where there are functional male soldiers. In the USA strain, female soldiers discriminate relative and non-relative reproductive larva, reducing the attack rates against the relatives^1^. Unfortunately, we have no data regarding male soldier aggression toward relatives and non-relatives in Japan. Note that the proportion of mixed broods in the wild in Japan is over 50% (usually 60% or higher), and that the sex ratio is very female biased (Table 3), as in the USA. Therefore, in the Japanese strain, the attack against conspecific female embryos (irrespective of relatives or not) should be always damaging the success of male owns. One plausible explanation is that the density of heterospecific competitors is high in the Japanese strain. Therefore, the benefit to kill heterospecific competitors outweighs the cost of killing the conspecific female embryos. This may also explain why the USA strain lost the defensive behaviors in sterile males, if heterospecific competitors are rare. We thus need the comparative data on heterospecific competitors and detailed life histories of the Japanese and USA strains.

Because the aggressiveness of the Japanese male soldiers is still kept, but lower than that of females, the functional males may be an adaptive response of a single unfertilized egg in a single host larva. Therefore, we expect that female wasps often lay a single unfertilized egg in a single host in the Japanese strain.

The existence of both functional and nonfunctional male soldiers in *C. floridanum* therefore suggests that the evolution of adaptive functions in male soldiers is likely to be affected by ecological conditions or oviposition traits. The evolution of functional male soldiers should be favored under the following conditions: (1) presence of numerous heterospecific competitors with high parasitization rates, (2) high frequency of single egg broods (especially unfertilized eggs), (3) high densities of adult wasps, as this would increase the likelihood of finding mates. This last condition, in particular, may not be satisfied in the American *Copidosoma* strain, resulting in mixed-sex broods in host larvae.

In the Japanese strain, the number of male soldiers is entirely determined genetically, while the female soldiers are epigenetically affected by environmental conditions^12^. That is, the male soldiers are expected to be more subjected to the pressure of natural selection. Assuming that having functional soldiers of both sexes is the primitive state, it is possible that soldiers may have evolved first in the Japanese population as adaptation against competing parasitoids. After that, this ancestral *Copidosoma* spread to North America, where, in response to mating success in mixed broods, male soldier function may have been lost secondarily in American *Copidosoma* strain. Soldier larvae of *C. floridanum* are unique among polyembryonic parasitoid wasps in that the level of male functions varies among geographical strains. Further research into the ecological conditions and genetic variability will clarify the developmental process of the male soldiers.

## Methods

### Insects

The larvae of *C. floridanum* occur as two morphs; a reproductive larval morph and sterile soldier larval morph^14^,^15^. The development and functions of both morphs have been well studied in *Copidosoma floridanum* (Hymenoptera: Encyrtidae). *C. floridanum* is a polyembryonic egg-larval parasitoid that is widely distributed in Asia and North America^16^. In *C. floridanum*, a single egg develop into a morula, which then proliferates clonally in the growing host larva and eventually produces more than 2000 clonal embryos^15^,^17^. Most of the embryos with germ cells develop into reproductive larvae in the final instar host larva, and eventually emerge as adult wasps. In addition to these embryos, a smaller number of embryos without germ cells develop precociously into structurally distinct slender larvae, which are referred to as soldier larvae^1^,^3^. Since the soldier larvae do not molt and finally die without pupating, they are completely sterile. *C. floridanum* produces clonal broods by laying either an unfertilised (haploid) egg that produces males or a fertilised (diploid) egg that produces females.

The polyembryonic parasitoid *C. floridanum* and its host *Ctenoplusia* ( = *Acanthoplusia) agnata* (Lepidoptera, Noctuidae) were collected in burdock fields in Tokyo, Japan, and maintained in the laboratory according to the methods described by Utsunomiya and Iwabuchi^15^. Natural parasitoid competitors of *C. floridanum*, *Glyptapanteles pallipes* (Braconidae), *Cotesia ruficrus* (Braconidae) and *Meteorus pulchricornis* (Braconidae) were also obtained from *C. agnata* larvae collected in the field in Tokyo and then reared together with a laboratory stock of *C. agnata*. *G.* *pallipes* and *C. ruficrus* are gregarious larval parasitoids of noctuid moths, and *M. pulchricornis* is a solitary larval parasitoid with an extremely wide host range. We also used *Cotesia glomerata* (Braconidae) as a factitious competitor of *C. floridanum*. *Cotesia glomerata* was obtained from the larvae of *Pieris rapae* collected in the field in Tokyo. Larvae (2nd instar) of *Pieris rapae* were presented to *C. glomerata* and the resultant parasitized hosts were maintained on an artificial diet.

### *In vitro* assay

*In vitro* assays were conducted in 35-mm plastic Petri dishes for six hours. One *C. floridanum* soldier larva from all-male (containing male soldiers) or all-female (containing female soldiers) was placed together with the 1st instar larva of a competitor species in a drop of 50 μl IPL-41 medium. As natural competitors, we used three larval parasitoids, *G. pallipes*, *C. ruficrus* and *M. pulchricornis*, which we obtained from larvae of the common host, *C. agnata*, collected in the field. As a factitious competitor, we used *C. glomerata*. The soldiers were continuously observed under a phase-contrast microscope during the assay period. If a soldier grasped a competitor larva with its mandibles for more than 1 min, then we recorded this as an aggressive behavior.

### *In vivo* contest

To determine whether the sex of *C. floridanum* affects the heterospecific competitive outcome, hosts were multiparasitized with each sex of *C. floridanum* and the other natural parasitoids. As in most hymenopteran species, unfertilized *C. floridanum* eggs produce males and fertilized eggs produce females. Since the oviposition behavior for unfertilized and fertilized eggs is different^17^, we were able to obtain single-sex broods derived from a single egg for each experiment. In the experiments, host eggs were parasitized by *C. floridanum* at 22–24 h post-oviposition. The parasitized hosts were then maintained until day 1 of the 2nd instar (L2D1), at which time they were allowed to be parasitized by other parasitoids. The multiparasitized hosts were then maintained until pupation, death or parasitoid emergence. One hundred hosts were used for each experiment, except for the competition with *M. pulchricornis* (n = 50).

### Field survey of sex ratios in mixed broods

To evaluate the effects of intraspecific competition on the resulting sex ratios in mixed broods, we conducted a field survey. For each mixed brood, we sexed one thousand emerging adults.

## Author Contributions

D.U., J.Y. and K.I. conceived the study. D.U. and A.T.N. performed experiments, D.U., J.Y. and K.I. wrote the manuscript.

## Figures and Tables

**Table 1:** The proportion of attacking *C. floridanum* individuals (%) and the time till initiating an attack (min) against four competitors in the in vitro competition experiment

Competitor	Sex of *C. f.*	No.	No. attacks (%)	χ^2^ test score p-value	Time till attack (min) (No. measured)	t-test score (df) p-value
*G. pallipes*	Male	29	15 (51.6) *	χ^2^ = 6.7579	265.5 ± 71.3(8)	t = 2.08(21)
	Female	30	25 (81.1)	p = 0.00933	189.3 ± 82.0(15)	P = 0.038
*C. ruficrus*	Male	49	15 (30.6)	χ^2^ = 8.9110	173.8 ± 94.3(15)	t = 2.08(28)
	Female	22	15 (68.1)	p = 0.00283	104.6 ± 56.1(15)	P = 0.023
*M. pulchricornis*	Male	27	14 (51.8)	χ^2^ = 4.703	191.3 ± 81.1(14)	t = 2.12(27)
	Female	18	15 (83.3)	p = 0.03011	91.9 ± 28.3(15)	p = 0.0003
*C. glomerata*	Male	49	14 (28.6)*	χ^2^ = 16.291	303.0 ± 45.3(12)	t = 2.05(25)
	Female	18	15 (83.3)	p = 0.00005	121.1 ± 74.8(15)	p = 0.0001

No. attacks indicates the number of individuals initiating attacking behavior in 6 hours in the total samples (No.). The differences of the number of attacking individuals between males and females of *C. f.* are tested by χ^2^ test (score and p-value are shown). In the time till attack, No. measured indicates the sample size. The time till attack between males and females of *C. f.* are tested by t-test (t-test score with degree of freedom and p-value are shown).

**Table 2:** Competitive ability of *C. floridanum* in singly and multi-parasitized hosts

		% of hosts producing					
Competitor multiparasitized	Sex of *C. floridanum*	Cf	Gp	Cr	Mp	% host pupation	% host death
*G. pallipes*	Male	68.0**^a^**	20.0**^b^**			4.0	8.0
	Female	78.0**^a^**	12.0**^b^**			2.0	8.0
*C. ruficrus*	Male	80.0**^a^**		6.0**^b^**		2.0	12.0
	Female	76.0**^a^**		4.0**^b^**		0.0	20.0
*M. pulchricornis*	Male	34.0**^a^**			0.0**^b^**	0.0	66.0
	Female	36.0**^a^**			2.0**^b^**	0.0	62.0
*C. floridanum* alone	Male	86.0				8.0	6.0
	Female	88.0				10.0	2.0
*G. pallipes* alone			80.0			8.0	12.0
*C. ruficrus* alone				74.0		14.0	12.0
*M. pulchricornis* alone					56.0	0.0	44.0

Hosts were multiparasitized at 2nd instar. One hundred hosts (n = 100) were used for each experiment, except for the competition with *M. pulchricornis* (n = 50). Two types of statistical tests are performed: (1) two-tailed binomial test by F-distribution approximation for the superiority of *C. floridanum* (male or female) against each competitor, where **a** indicates significant at p = 0.00000 and (2) Fisher's exact probability test for the effects of *C. floridanum* (male or female) on the success of each competitor, where **b** indicates significant at p = 0.00000.

**Table 3:** The sex ratio of mixed broods in *C. floridanum *collected in the field in Japan

Year	No. Broods	Average male ratio	Ranges of male ratio (max-min)
2003	20	0.031	0.049−0.008
2004	21	0.027	0.056−0.007
2005	26	0.057	0.132−0.002
2007	25	0.036	0.076−0.005

In each brood, 1000 adults are sexed. One-tailed binomial tests indicate that male ratios are significantly lower in each year at p = 0.00000.

## References

[b1] Grbic M., Ode P. J. & Strand M. R. Sibling rivalry and brood sex rations in polyembryonic wasps. Nature 360, 254–256 (1992).

[b2] Smith M. S., Milton I. & Strand M. R. Phenotypically plastic traits regulate caste formation and soldier function in polyembryonic wasps. J. Evol. Biol. 23, 2677–2684 (2010). 21040070 10.1111/j.1420-9101.2010.02127.x PMC3057481

[b3] Harvey J. A., Corley L. S. & Strand M. R. Competition induces adaptive shifts in caste ratios of a polyembryonic wasp. Nature 406, 183–186 (2000). 10910357 10.1038/35018074

[b4] Giron D., Dunn D. W., Hardy I. C. W. & Strand M. R. Aggression by polyembryonic wasp soldiers correlates with kinship but not resource competition. Nature 430, 676–679 (2004). 15295600 10.1038/nature02721

[b5] Giron D., Harvey J. A., Johnson J. A. & Strand M. R. Male soldier caste larvae are non-aggressive in the polyembryonic wasp *Copidosoma floridanum*. Biol. Lett. 3, 431–434 (2007). 17535791 10.1098/rsbl.2007.0199 PMC2390675

[b6] Gardner A., Hardy I. C. W., Taylor P. D. & West S. A. Spiteful soldirs and sex ratio conflict in polyembryonic parasitoid wasps. Am. Nat. 169, 519–533 (2007). 17427122 10.1086/512107

[b7] Ode P. J. & Strand M. R. Progeny and sex allocation decisions of the polyembryonic wasp *Copidosoma floridanum*. J. Anim. Ecol. 64, 213–224 (1995).

[b8] Grbic M., Rivers D. & Strand M. R. Caste formation in the polyembryonic wasp *Copidosoma floridanum* (Hymenoptera: Encyrtidae): In vivo and in vitro analysis. J. Insect Physiol. 43, 553–565 (1997). 12770418 10.1016/s0022-1910(97)00004-8

[b9] Yamamoto D., Henderson R., Corley L. S. & Iwabuchi K. Intrinsic, inter-specific competition between egg, egg-larval, and larval parasitoid of plusiine loopers. Ecol. Entomol. 32, 221–22 (2007).

[b10] Segoli M., Kaesar T., Harari A. R. & Bouskila A. Limited kin discrimination abilities mediate torelance toward relatives in polyembryonic parasitoid wasps. Behav. Ecol. 20, 761–767 (2009).

[b11] Segoli M., Bouskila A., Harari A. R. & Kaesar T. Developmental patterns in the polyembryonic parasitoid wasp *Copidosoma koehleri*. Anthropod Structure and Development 38, 84–90 (2009). 10.1016/j.asd.2008.05.003 18638571

[b12] Watanabe K., Nishide Y., Roff D. A., Yoshimura J. & Iwabuchi K. Environmental and genetic controls of soldier caste in a parasitic social wasp. Sci. Rep. 2, 729 (2012). 23087811 10.1038/srep00729 PMC3476458

[b13] Giron D., Ross K. G., & Strand M. R. Presence of soldier larvae determines the outcome of competition in a polyembryonic wasp. J. Evol. Biol. 20, 165–172 (2007). 17210009 10.1111/j.1420-9101.2006.01212.x

[b14] Strand M. R. & Grbic M. The development and evolution of polyembryonic insects. In: Current Topics in Developmental Biology, (Pederson, R. A. & Schatten, G. P., eds), Vol. 35, pp. 121–159 (Academic Press, New York, 1997). 9292269 10.1016/s0070-2153(08)60258-6

[b15] Utsunomiya A. & Iwabuchi K. 2002. Interspecific competition between the polyembryonic wasp *Copidosoma floridanum* and the gregarious endoparasitoid *Glyptapanteles pallipes*. Ent. exp. appl. 104, 353–362 (2002).

[b16] Noyes J. S. Copidosoma truncatellum (Dalman) and *C. floridanum* (Ashmead) (Hymenoptera, Encyrtidae), two frequently misidentified polyembryonic parasitoids of caterpillars (Lepidoptera). System. Entomol. 13, 197–204 (1988).

[b17] Strand M. R. Development of the polyembryonic parasitoid *Copidosoma floridanum* in *Trichoplusia ni*. Ent. exp. appl. 50, 7–47 (1989).

